# Linking neural circuits to the mechanics of animal behavior in *Drosophila* larval locomotion

**DOI:** 10.3389/fncir.2023.1175899

**Published:** 2023-08-17

**Authors:** Hiroshi Kohsaka

**Affiliations:** ^1^Graduate School of Informatics and Engineering, The University of Electro-Communications, Chofu, Tokyo, Japan; ^2^Department of Complexity Science and Engineering, Graduate School of Frontier Science, The University of Tokyo, Chiba, Japan

**Keywords:** *Drosophila*, locomotion, motor circuits, kinematics, kinetics, exploration, interspecific diversity

## Abstract

The motions that make up animal behavior arise from the interplay between neural circuits and the mechanical parts of the body. Therefore, in order to comprehend the operational mechanisms governing behavior, it is essential to examine not only the underlying neural network but also the mechanical characteristics of the animal’s body. The locomotor system of fly larvae serves as an ideal model for pursuing this integrative approach. By virtue of diverse investigation methods encompassing connectomics analysis and quantification of locomotion kinematics, research on larval locomotion has shed light on the underlying mechanisms of animal behavior. These studies have elucidated the roles of interneurons in coordinating muscle activities within and between segments, as well as the neural circuits responsible for exploration. This review aims to provide an overview of recent research on the neuromechanics of animal locomotion in fly larvae. We also briefly review interspecific diversity in fly larval locomotion and explore the latest advancements in soft robots inspired by larval locomotion. The integrative analysis of animal behavior using fly larvae could establish a practical framework for scrutinizing the behavior of other animal species.

## Introduction

**FIGURE 1:**
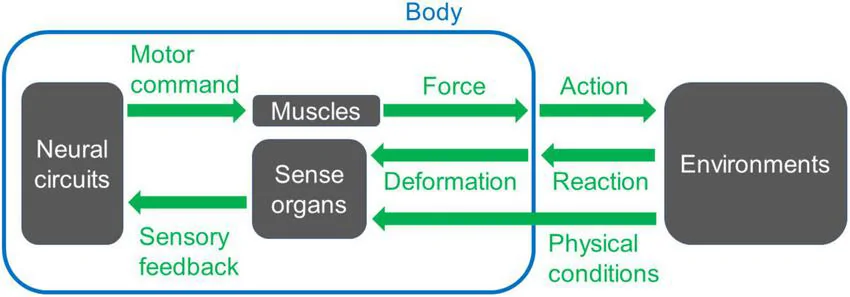
A basic scheme of a system for animal movement. Neural circuits send signals to muscles to generate forces to move the body. The motion induces interaction with the environments and reaction from them. The reaction can trigger deformation or movement of the body, which is detected by sense organs. Other physical conditions of the environments are also monitored by sense organs, and the information for each sensory modality feeds back to the neuronal circuits that create the movement. When appropriate spatiotemporal action is operated, the animal can perform locomotion, i.e., displace its body position within the surrounding space.

The nervous system works together with the body and its environment to produce behavior (Schmidt-Nielsen, 1997; Alexander, 2006; Biewener and Patek, 2018; Figure 1). Neural circuits for motor control, including central pattern generators, create spatiotemporal patterns of neural activity (Marder and Calabrese, 1996; Marder and Bucher, 2001; Grillner, 2006; Grossmann et al., 2010; Selverston, 2010; Grillner and El Manira, 2020). These neural signals are delivered to motor neurons, which innervate the muscles. At the neuromuscular junction, where motor commands are conveyed to the muscles, a series of neural signals is transformed into a temporal pattern of force production (Sandow, 1952; Caille et al., 1985; Peron et al., 2009; Calderón et al., 2014; Shishmarev, 2020; Ormerod et al., 2022). This physiological process occurs at every neuromuscular junction, resulting in the generation of a spatiotemporal pattern of muscular force throughout the body. The coordinated pattern determines which muscles contract, their timing, and the strength of contraction. The forces exerted on part of the body initiate movement within the physical constraints imposed by the body, including material properties such as bone solidity and joint flexibility, as well as external forces such as friction at the foot-substrate interface (Ferretti et al., 2011; McHenry, 2012; Nichols et al., 2014; Labonte and Federle, 2015; Godoy-Diana and Thiria, 2018; Sylvester et al., 2021). Each movement is detected and monitored through sensory feedback provided by afferent nerves, which can be utilized to refine the final motor output (Garcia-Campmany et al., 2010; Kiehn, 2016; Nielsen, 2016; Kaplan et al., 2018; Mantziaris et al., 2020; Mongeau et al., 2021; Figure 1). Consequently, animal behavior emerges from a dynamic interplay between neural circuits, muscles, sensory neurons, and the surrounding environments.

The signal flow within motor control circuits can give rise to various complexities. In particular, the conversion of neural signals into body movement introduces several intricacies: 1. From the neural circuit level [cf. ∼86 billion neurons in the human brain (Azevedo et al., 2009)] to the body level (cf. hundreds of muscles, bones, and joints in the adult human body), the degrees of freedom are reduced. During this step, information processed within the circuit needs to be compressed in accordance with the body’s structural constraints (De Groote and Falisse, 2021; Kearney et al., 2022). Furthermore, the motor system exhibits high redundancy so that multiple muscle activation patterns can generate the same movement (Prilutsky, 2000; Song, 2020). For instance, modeling studies have demonstrated that distinct spatiotemporal patterns of muscle activities can produce identical motions in stereotyped human behaviors such as walking (Crowninshield and Brand, 1981; Collins, 1994) and running (Miller R. H. et al., 2012). One possible rationale behind this redundancy would be the fact that the co-activation of antagonistic muscles may not significantly impact overall kinematics (Collins, 1994). 2. The firing rate of motor neurons and the resulting muscular contraction force display a non-linear relationship (Caille et al., 1985; Durfee, 1993). Non-linearity within the motor control system can cause a variety of phenomena, including resonance, compression-tension asymmetry, anisotropy in passive muscle properties, and time-dependent kinematic properties (such as hysteresis) (Solomonow, 2006; Ting and McKay, 2007; Tytell et al., 2014; Strogatz, 2015; Hashemi et al., 2020). 3. The energy in the body is not conserved. Elastic components, such as tendon cells, dissipate kinetic energy from muscle contraction (Alexander, 2006; McHenry, 2012; Roberts, 2016; Werkhausen et al., 2017). 4. As exemplified in multi-joint movement, the motion of body elements is tightly constrained by musculoskeletal properties (Prilutsky, 2000; D’Avella et al., 2003; Carson and Kelso, 2004; Biryukova and Sirotkina, 2020). These complexities – reduction of degrees of freedom, non-linearity, energy dissipation, and constraints from body configurations – can influence the final outcome of body movements. Consequently, in investigating the underlying mechanisms of animal behavior, both the mechanical properties of the body and the patterns of neural signals need to be considered (Chiel and Beer, 1997; Nishikawa et al., 2007; Tytell et al., 2011; Miller L. A. et al., 2012; Ting et al., 2015; Gomez-Marin and Ghazanfar, 2019). Thus, an integrative approach targeting neural networks and body mechanics is essential.

This review introduces *Drosophila* larval locomotion as a valuable model system for studying the neuromechanics of animal behavior. Firstly, we highlight the spatiotemporal pattern and mechanical aspects of larval crawling motion. Subsequently, we provide a brief overview of the relevant neural circuits involved in larval crawling and exploration. Finally, we discuss future directions for utilizing fly larvae as a model system to explore interspecific diversity in animal locomotion and to develop animal-inspired soft robots.

## The *Drosophila* larval motor systems

### *Drosophila* larval locomotion

Fly larvae exhibit a variety of behaviors, including crawling (which is the primary focus of this article), turning, head sweeping, rolling, digging, and self-righting (Green et al., 1983; Picao-Osorio et al., 2015; Kim et al., 2017). Of particular interest to researchers are the neural mechanisms and muscle kinematics underlying rolling behavior, which are currently being investigated in detail (He et al., 2022; Cooney et al., 2023). How to generate multiple behavioral patterns using the same neural circuit is one of the fundamental questions in neuroscience (Briggman and Kristan, 2008), and larval motor circuits can provide an excellent model system for studying this phenomenon (Kohsaka et al., 2019; Zarin et al., 2019; Hiramoto et al., 2021; Huang and Zarin, 2022). While various larval behaviors exist, this review specifically focuses on larval locomotion, as its neural and physical mechanisms have been extensively examined.

**FIGURE 2:**
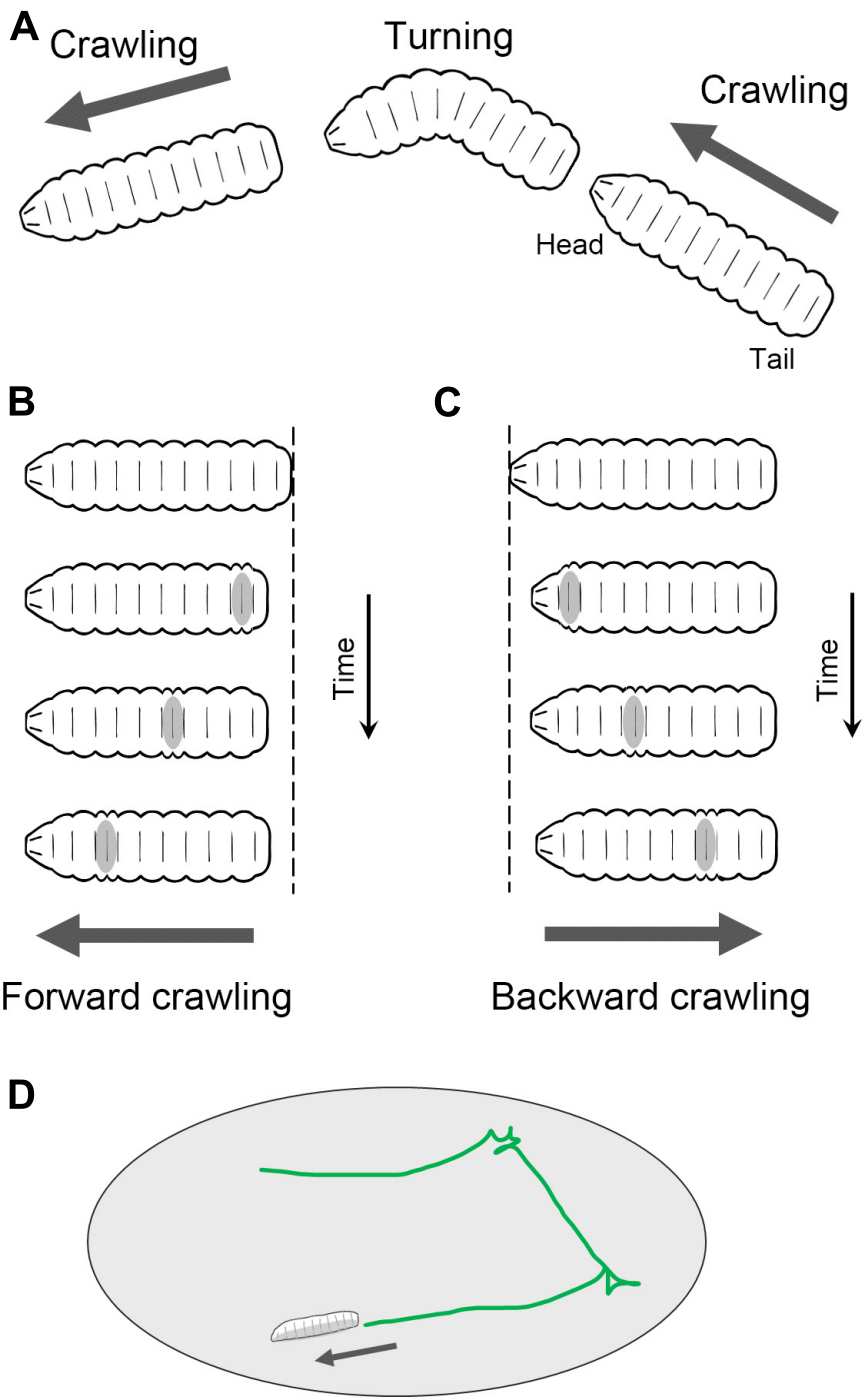
Locomotion of *Drosophila* larvae. **(A)** Fly larvae locomote approximately straight by peristaltic crawling and change the direction of the crawling by turning. In addition, larvae crawl backward occasionally. **(B)** In forward crawling, segmental contraction propagates from the posterior to anterior segments. **(C)** In backward crawling, segmental contraction propagates from the anterior to posterior segments. **(D)** By switching between forward crawling, turning, and backward crawling, larvae navigate on a two-dimensional surface.

Fly larvae navigate on a two-dimensional surface through crawling and turning movements (Figure 2A). Forward crawling is generated by sequential segmental contractions from the posterior to anterior segments (Figure 2B), while backward crawling is generated in the opposite direction, from the anterior to posterior (Figure 2C). Although the crawling direction can be gradually altered during locomotion, sharp changes in direction are achieved through turning behavior. Turning is accomplished by unilateral body bending followed by peristaltic motions (Lahiri et al., 2011). The combination of these movements, such as forward crawling, backward crawling, and turning, enables larvae to move in a two-dimensional space facilitating food foraging and the search for suitable molting sites (Figure 2D; Green et al., 1983; Kohsaka et al., 2017; Clark et al., 2018).

Crawling is a form of axial locomotion that involves the propagation of muscle activity along the length of the body. Such sequential (or metachronal) waves are generated by propagation of neural activity that travels through the central nervous system (CNS). One fundamental research question in the study of axial locomotion is the coordination of neural activity between segments. Traveling waves of neural activity underlying axial locomotion are observed and examined in diverse species, including leeches, crayfish, lampreys, zebrafish, tadpoles, and mice (Falgairolle et al., 2013). Fly larvae provide a valuable model for studying the neural circuit and body mechanics involved in axial locomotion. With their relatively simple nervous system and the availability of diverse experimental tools, they offer a promising platform for studying the intricate mechanisms underlying axial locomotion. Through the examination of both the neural circuitry and biomechanics in fly larvae, researchers can uncover the fundamental principles and coordinated processes that govern axial locomotion, shedding light on an essential aspect of locomotion.

### *Drosophila* larval neuromuscular systems

**FIGURE 3:**
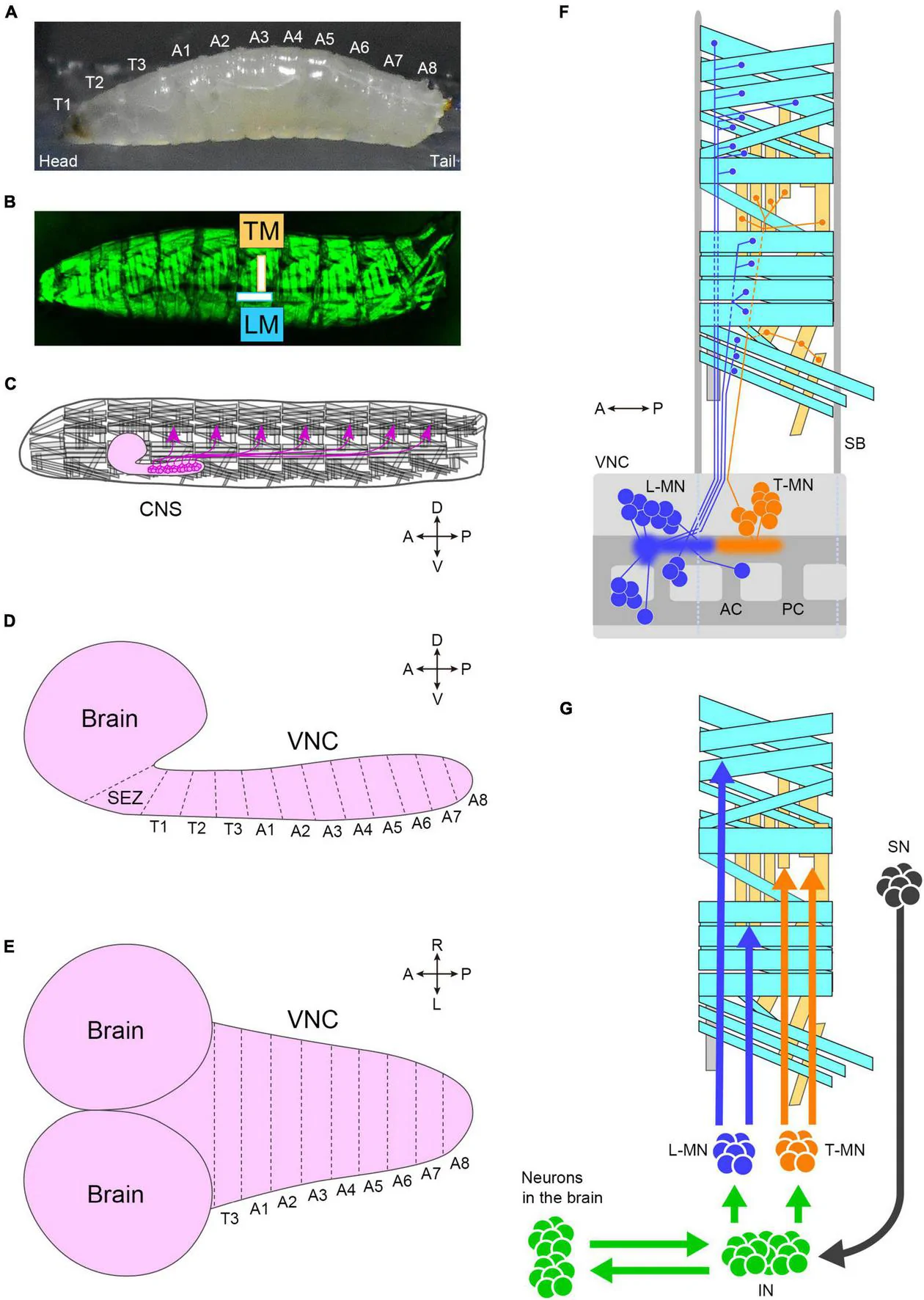
The neuromuscular system of fly larvae. **(A)** A side view photo of a third instar larva. **(B)** GFP-labeled body-wall muscles in a third instar larva. **(C)** A schematic of the larval motor system. Motor neurons (magenta) locate in the CNS and innervate body-wall muscles. The segmental layout of the larval CNS from the lateral **(D)** and dorsal **(E)** view. **(F)** Motor neurons and muscles in a hemisegment. Cyan boxes present longitudinal muscles, and yellow boxes present transverse muscles. Blue neurons (L-MN: motor neurons targeting longitudinal muscles) innervate longitudinal muscles. Orange neurons innervate transverse muscles (T-MN: motor neurons targeting transverse muscles). Discs in the VNC indicate the cell body of motor neurons, and sprayed regions indicate the dendritic regions of motor neurons. The demarcation of dendrites between L-MN and T-MN exhibits the myotopic map. **(G)** Neurons involved in motor control. Tn, the *n*-th thoracic segment; An, the *n*-th abdominal segment; TM, transverse muscles; LM, longitudinal muscles; CNS, the central nervous system; SEZ, the subesophageal zone; VNC, the ventral nerve cord; A, anterior; P, posterior; D, dorsal; V, ventral; R, right; L, left; L-MN, motor neurons targeting longitudinal muscles; T-MN, motor neurons innervating transverse muscles; SB, segment boundary; AC, anterior commissure; PC, posterior commissure; SN, sensory neuron; IN, interneuron. Source images of **(A)** and **(B)**: Kohsaka et al., 2019.

The peristaltic larval crawling is based on the segmented structure of the larval body. The external observation reveals three thoracic and eight abdominal segments (Figure 3A). During larval crawling, both sides of a segment contract simultaneously, while in the bending motion, only one side of the body undergoes contraction. Each of the left and right hemisegments contains approximately 30 body wall muscles, including longitudinal and transverse muscles (Keshishian et al., 1996; Figure 3B). The longitudinal muscles extend along the head-to-tail body axis within a single segment (indicated by “LM” in Figure 3B), while the transverse muscles span perpendicular to the body axis (indicated by “TM” in Figure 3B). This musculature layout is repeated throughout the majority of the thoracic and abdominal segments (Figure 3B).

Imaging studies utilizing GFP-labeled muscles have provided insights into the distinct activity patterns of longitudinal and transverse muscles during crawling. In larval crawling within a fabricated channel, the contraction of longitudinal muscles precedes that of transverse muscles (Heckscher et al., 2012). These muscle activation patterns have been observed at the single-cell resolution, which reveals that the muscles are grouped into four co-active populations (Zarin et al., 2019). Furthermore, a recent investigation analyzing muscle kinematics in larvae crawling on an open surface demonstrates that the contraction of longitudinal muscles propagates from one end to the other while transverse muscles contract between peristaltic waves (Liu et al., 2022). The specific role of transverse muscles in crawling remains unclear, but they may potentially antagonize longitudinal contractions and/or regulate the timing of crawling repetition.

The generation of spatiotemporal patterns of muscle contraction for locomotion in fly larvae is controlled by the CNS, which consists of the brain, subesophageal zone (SEZ), and ventral nerve cord (VNC; the center for all local neuronal control of locomotion) (Figures 3C–E). The estimated number of neurons in the CNS in the first instar is approximately 10,000 (Scott et al., 2001), about 8,000 of which are located in the VNC (Avalos et al., 2019). Similar to the body wall, the VNC is segmented into three thoracic segments (T1 to T3) and eight abdominal neuromeres (A1 to A8) (Figures 3D, E). The boundaries between neighboring neuromeres may not be clearly defined due to the compression of the VNC during development, as observed in certain arthropods (Smarandache-Wellmann, 2016).

**FIGURE 4:**
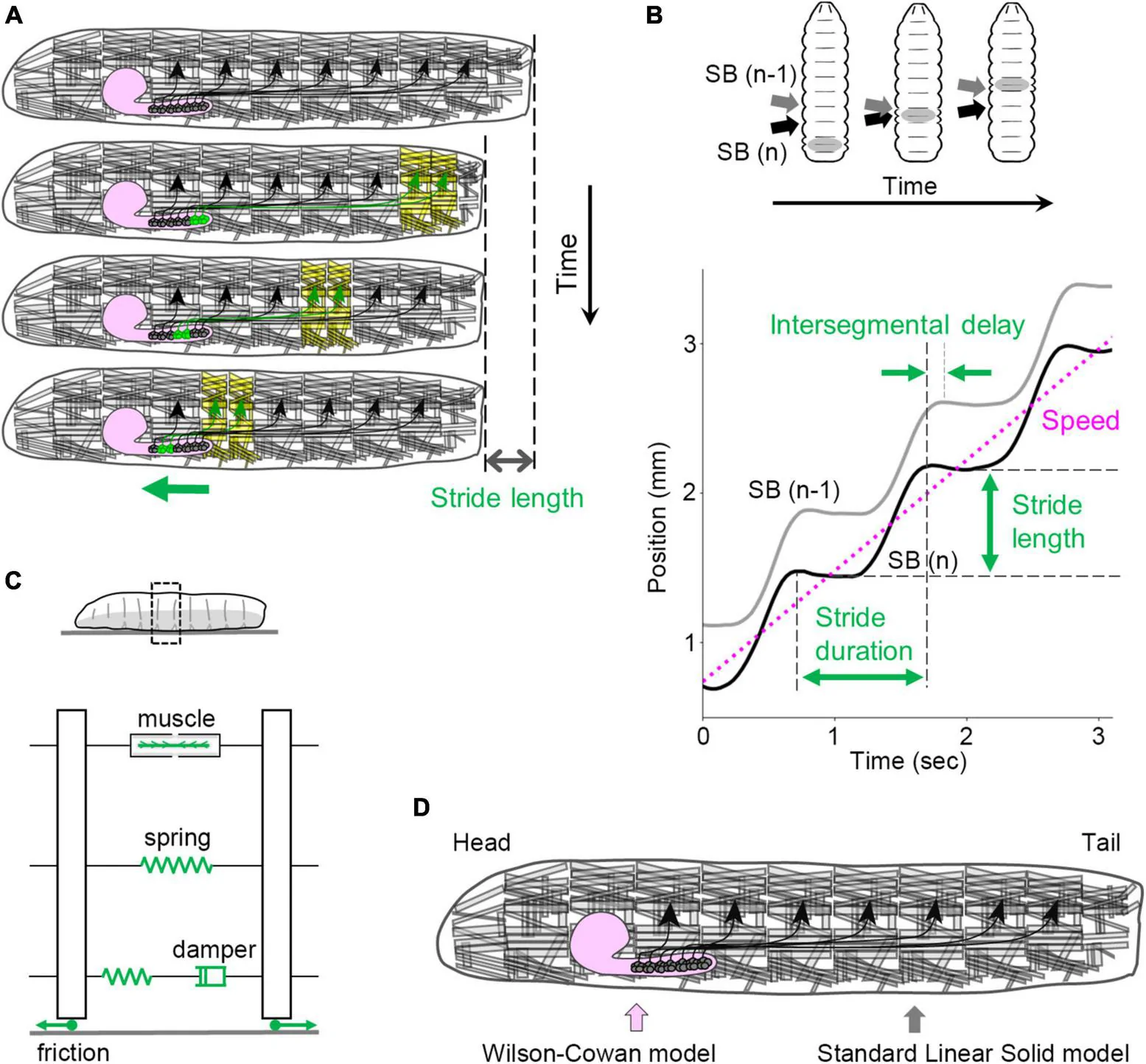
Kinematics and kinetics of larval crawling. **(A)** In peristaltic crawling, neural activity propagates within the VNC (green), and muscular contraction propagates from posterior to anterior segments in the body wall. **(B)** Kinematic quantities in larval crawling. (Top) The positions of segment boundaries are displaced during crawling. Gray arrows mark the (*n* – 1)th segment boundary, and black arrows mark the *n*-th segment boundary. (Bottom) Kinematic quantities extracted from the traces of segment boundaries. **(C)** A standard linear solid model of the larval body and the interaction with the substrate. **(D)** In the integrated neuromechanical model, the CNS is simulated by the Wilson–Cowan model, while the body wall is simulated by the standard linear solid model represented in panel **(C)**. SB, segment boundary.

Each neuromere contains approximately 73 motoneurons (∼35 unilateral neurons on each side and 3 bilateral neurons; Landgraf et al., 1997; Schmid et al., 1999). Motoneurons extend their axons through one of the three peripheral nerves: the segmental, intersegmental, or transverse nerves (Thomas et al., 1984; Landgraf et al., 1997). The dendrites of motor neurons are demarcated according to their target muscles to form a myotopic map (Landgraf et al., 2003; Kim et al., 2009; Figure 3F). Motor neurons are classified into four groups based on the position and orientation of their target muscles [in Figure 3F, the three groups targeting longitudinal muscles are merged into a single group (L-MN)]. The myotopic map reveals the segregation of dendrites of distinct motor groups within the CNS, potentially contributing to the layout of the premotor interneuron network. The connectivity between premotor interneurons and motor neurons has been extensively characterized, and the firing patterns in fictive motion (neural activity exhibiting wave propagation along the head-to-tail axis in an isolated CNS) have been investigated in detail (Zarin et al., 2019). The anteroposterior propagation of neural activity within the VNC underlies peristaltic locomotion (see Figure 4A).

Sensory neurons in the body wall are classified into different classes based on their location and morphology. Class I multidendritic neurons are proprioceptors and are involved in controlling locomotion speed (Caldwell et al., 2003; Hughes and Thomas, 2007; Song et al., 2007; Cheng et al., 2010; Singhania and Grueber, 2014; He et al., 2019; Vaadia et al., 2019). The proprioceptor neurons possess specific sensing properties to monitor the deformation of the body wall, and the information about the body movement is transmitted to the CNS (He et al., 2019; Vaadia et al., 2019). It has also been observed that asymmetric morphology of the proprioceptor neurons along the body axis correlates with the sensitivity to the direction of larval crawling: sensory neurons with dendrites projecting anteriorly are sensitive to backward crawling, while those with dendrites projecting posteriorly are sensitive to forward crawling (He et al., 2019).

The brain and VNC are connected by multiple neuronal fiber bundles (Cardona et al., 2009). A recent study has shown that descending neurons form synapses with only a small fraction of premotor and pre-premotor neurons (Winding et al., 2023). This suggests that the VNC has the capability to generate motor patterns by itself, which is consistent with the observation that the brain is not essential for wave propagation of neural activity in the fly VNC (Berni et al., 2012; Pulver et al., 2015; Matsunaga et al., 2017) and other arthropod locomotion circuits (North and Greenspan, 2007; Hooper and Buschges, 2017).

In summary, in the neuromuscular systems of *Drosophila* larvae, the muscles, motor neurons, and sensory neurons are well characterized. The accumulation of knowledge and techniques has provided a solid foundation for studying the physiological and developmental characteristics of these cell types. Furthermore, as described below, understanding the neural mechanisms of larval motor control relies on comprehensive knowledge of the peripheral systems.

## Mechanics of larval locomotion

### Kinematics of larval locomotion

Mechanics is a field in physics that studies the motion of objects. It encompasses two fundamental concepts: kinematics and kinetics. Kinematics focuses on describing the movement of objects within a given frame of reference, while kinetics deals with the forces acting on those objects. In the context of larval locomotion, kinematics can be examined by tracing the position of the segment boundaries during crawling.

The peristaltic crawling of fly larvae is generated by the propagation of segmental contractions from one end to the other (Figure 4A). To analyze the kinematics of this motion, several parameters can be defined (Figure 4B). The stride length refers to the displacement of the body in a single stride, which corresponds to one step forward or backward. The stride duration represents the time required for a single stride. By analyzing the time series data of the body position (which can be quantified by the centroid coordinates), the speed (a scalar variable), or velocity (a vector variable) can be calculated. During a peristaltic wave, the longitudinal muscles in each segment contract and relax once, leading to changes in the length of each segment. These changes can be described by the maximum and minimum segment lengths. For the temporal aspect, the duration between the contraction and relaxation in a single peristaltic wave is referred to as the contraction duration. In addition, the delay in contraction between neighboring segments can be quantified as the intersegmental delay. These measures provide quantitative information about the kinematics of larval crawling (Sun et al., 2022).

In the framework of mechanics, the kinematics of objects can be obtained by solving equations of motion, which are typically described by a set of differential equations. To establish the equations of motion for larval locomotion, several factors need to be considered, including the type of mechanics involved, neural signaling, muscular forces, and material properties of the body. Understanding these aspects is crucial for studying the mechanical principles underlying larval locomotion and its relationship with neural control.

### Mechanical description of larval locomotion

Given the physical characteristics and scale of larval locomotion, Newtonian classical mechanics is a suitable framework for describing its mechanical behavior. The speed of larval crawling, on the order of 10^–3^ m/s, makes relativistic effects negligible in the context of larval locomotion. Additionally, the size of larvae, on the order of 10^–3^ m, is significantly larger than their de Broglie wavelength, which is on the order of 10^–24^ m. Consequently, quantum mechanics effects can be considered negligible in relation to larval behavior (Landau and Lifshitz, 1976; Feynman et al., 1977; Goldstein, 1980). Several laws of classical mechanics are applicable to a larval movement. Newton’s second law states that the acceleration of a body is proportional to the total force exerted on it. This law is relevant for understanding the relationship between forces and acceleration in larval locomotion. Newton’s third law describes the equal and opposite reaction forces between interacting objects, which is important for understanding the interaction between larvae and the surface substrate they crawl on (Trueman, 1975). Hooke’s law, a phenomenological law, states that the tensile force (stress) in an elastic material is linearly proportional to the deformation (strain). This law is applicable for modeling the flexible nature of the larval body during locomotion.

Considering the neural control of larval locomotion, the CNS plays a crucial role in delivering spatiotemporal neural signals to muscles. Since the motor circuits responsible for larval crawling have not been fully characterized at the single-cell scale, modeling the CNS requires a level of abstraction. Some successful models have represented the VNC as a linear chain of neuromeres, with each neuromere simulated by a Wilson–Cowan model (Wilson and Cowan, 1972) consisting of excitatory and inhibitory neuronal populations (Gjorgjieva et al., 2013). This approach has been able to generate peristaltic waves in both forward and backward directions (Gjorgjieva et al., 2013; Pehlevan et al., 2016; Sun et al., 2022).

In the study of larval locomotion kinetics, the forces generated by muscular contractions and frictional forces are important considerations. The forces generated by spontaneous or electrically- or optogenetically induced muscular contractions have been quantified using force transducers (Paterson et al., 2010; Ormerod et al., 2022; Sun et al., 2022). The measurements have shown variability in the magnitude of the force from measurement to measurement, but the maximum contraction force exerted by the whole larval body is estimated to be around 7 mN (Ormerod et al., 2022; Sun et al., 2022). In addition, a recent study using optical methods has measured the reaction forces between larvae and the agarose substrate they crawl on, ranging from 1 to 7 μN (Booth et al., 2022). These quantitative analyses contribute to our understanding of the physical forces involved in larval crawling.

To fully describe the mechanics of larval locomotion, the physical properties of the larval body are essential parameters. In materials science, mechanical properties are often measured using stress-relaxation tests, which involve extending a specimen quickly and measuring the time-dependent reduction in stress under constant strain (Banks et al., 2011). Similar tests have been performed on fly larvae, revealing their passive elasticity and viscosity and the standard linear solid model is suitable compared with other spring-and-damper models (Figure 4C; the elasticity of the whole body is ∼4.5 mN/mm, and the viscosity of the whole body is ∼240 mN s/mm) (Sun et al., 2022). Although the non-linear properties of the larval body were not fully explored in these studies, a linear approximation of the larval body provides a useful standpoint for mechanical descriptions.

By integrating the neuronal and mechanical formulations described above, qualitative or quantitative physical models of larval locomotion can be developed. A few neuromechanical models are introduced below.

### Integrated and other neuromechanical models of larval locomotion

The development of neuromechanical models for larval crawling has provided valuable insights into the mechanisms of locomotion. One pioneering work has represented the CNS as a linear chain of the Wilson–Cowan models and incorporated a mechanical model with a spring and a damper for each segment (Pehlevan et al., 2016). The elasticity and viscosity were inferred theoretically. The integrated model successfully generates peristaltic crawling that resembles larval locomotion qualitatively and has made a prediction regarding the coupling between neuromeres through proprioception. This prediction suggests that the deformation of the body and the resulting activation of proprioceptors are sufficient to convey the activity from one end to the other end of the VNC. If this is the case, the segmental activity would be propagated by two parallel mechanisms, body-aided mechanical waves and CNS-derived traveling waves since the isolated CNS can generate propagation of neural activity without any sensory feedback (see section “Population circuit activity patterns for locomotion”). While challenging to test experimentally, future advancements in genetic tools may allow for the verification of this prediction.

Another quantitative neuromechanical model has been developed based on measurements of the physical properties of larvae, particularly their passive elasticity and viscosity (Figure 4C; Sun et al., 2022). By integrating these measured values into the model, it is able to reproduce the kinematics of larval crawling quantitatively (Figure 4D). This model successfully reproduces previous experimental results with optogenetic perturbation (Inada et al., 2011; Kohsaka et al., 2014). Furthermore, the model predicts that several kinematic parameters of crawling, such as contraction duration and intersegmental delay, are not affected in low friction conditions. The model’s prediction was validated through experimental measurements of larval crawling under low-friction conditions (Sun et al., 2022). These observations indicate the significance of force measurements and material properties in improving the quantitative description of larval behavior.

Besides the Wilson–Cowan model and the springs-and-dampers approach, other physical formulations have been proposed. For instance, a mechanical model incorporating damped translational and torsional springs has demonstrated that local sensory-motor reflexes and long-range mutual inhibition between reflexes in distant segments are sufficient to generate crawling behavior (Loveless et al., 2019). Additionally, an effective field theory with a quadratic Hamiltonian has been successful in describing larval movements, including crawling (Loveless et al., 2021). These alternative theoretical approaches offer novel perspectives on larval locomotion and have the potential to uncover fundamental principles underlying it.

To sum up, a combination of theoretical and experimental approaches contributes to the quantitative understanding of the kinematics of larval locomotion. By incorporating experimentally revealed neural circuits into neuromechanical models, it will become possible to describe the neural and physical foundations of larval locomotion in a quantitative manner in the future. Furthermore, these neuromechanical models can serve as valuable tools for assessing the roles of interneurons in larval motor control.

## Neural circuits for larval locomotion

### Experimental approaches to characterize interneurons for larval behavior

The study of neural circuits controlling *Drosophila* larval locomotion has witnessed significant advancements in the past decade, mainly thanks to two critical approaches: genetic labeling and connectomics. These approaches have provided valuable insights into the functioning of neural circuits involved in larval locomotion.

Genetic labeling techniques, such as the Gal4-UAS system, LexA-LexAop system, and Q system, have been instrumental in targeting specific neurons and expressing genes that enable the manipulation and observation of neural activity (Brand and Perrimon, 1993; Lai and Lee, 2006; Luan et al., 2006; Venken et al., 2011; Jenett et al., 2012; Manning et al., 2012; Li et al., 2014; Bellen et al., 2015; Riabinina et al., 2015, 2019). With these tools, researchers can express a variety of effector genes such as calcium ion sensors to monitor neuronal activity, membrane proteins for sustained perturbation of neural activity, RNA interference genes for gene silencing, and optogenetics tools for transient manipulation of neural activity using visible light (Dietzl et al., 2007; Perkins et al., 2015; Simpson and Looger, 2018). By selectively targeting specific neurons, researchers can examine their roles in larval locomotion.

Connectomics, the comprehensive study of neural connectivity, has also contributed significantly to our understanding of larval locomotion. The wiring diagrams of neural circuits in first-instar (Ohyama et al., 2015; Schneider-Mizell et al., 2016) and third-instar larvae (Gerhard et al., 2017) have been examined, mapping out the connections between neurons. Worldwide collaborative efforts have allowed for the study of various neural circuits involved in feeding (Miroschnikow et al., 2018), escaping (Ohyama et al., 2015; Burgos et al., 2018; Imambocus et al., 2022), learning and memory (Eichler et al., 2017; Saumweber et al., 2018; Eschbach et al., 2020), vision (Larderet et al., 2017), chemotaxis (Tastekin et al., 2018; Vogt et al., 2021), and locomotion (Kohsaka et al., 2017; Clark et al., 2018; Gowda et al., 2021). By deciphering the connectivity patterns and identifying key neurons and their interactions, the neural mechanisms that drive larval locomotion have been partially clarified and will be further revealed in the future.

The same approaches in studying neural circuits with genetic resources and connectomics are applicable in the study of adult flies (Griffith, 2012; Coen and Murthy, 2016; Dubowy and Sehgal, 2017; von Reyn et al., 2017; Guo et al., 2022; Namiki et al., 2022; Currier et al., 2023). These techniques have been employed to investigate neural circuits involved in adult behaviors such as flight, escape, courtship, feeding, aggressive, and circadian behaviors.

In summary, the combination of genetic labeling and connectomics techniques has significantly advanced the study of larval motor circuits at the single-cell level. Several interneurons identified by these powerful tools are overviewed in the next section.

### The central nervous system for larval locomotion

**FIGURE 5:**
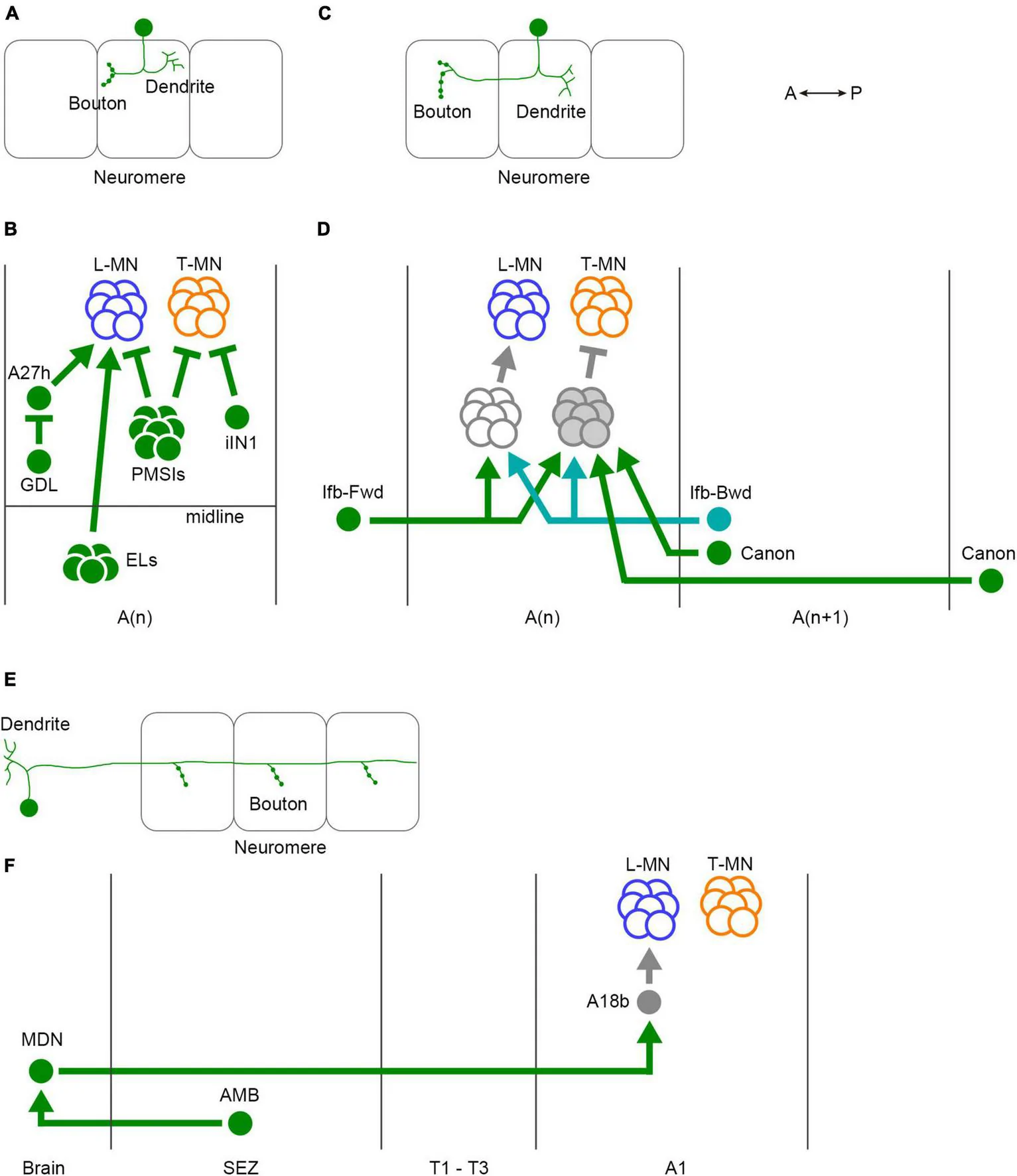
Three classes of interneurons for regulation of larval crawling. **(A)** The neurites of intrasegmental neurons are confined within a single neuromere. **(B)** Intrasegmental interneurons identified in the larval motor circuits. **(C)** Intersegmental neurons convey the activity between neuromeres. **(D)** Intersegmental interneurons identified in the larval motor circuits. **(E)** Ascending and descending neurons are involved in the communications between the VNC and the brain. **(F)** Ascending and descending neurons identified in the fly larval motor circuits. In panels **(A,C,E)**, one neuron and three neuromeres are shown. Filled circles in the VNC represent presynaptic terminals. Branches indicate dendrites. L-MN, motor neurons targeting longitudinal muscles; T-MN, motor neurons innervating transverse muscles; SB, segment boundary; PMSIs, period-positive median segmental interneurons; iIN, inhibitory interneuron; GDL, GABA-positive dorsal lateral neuron; ELs, Eve-positive lateral neurons; Ifb-Fwd, intersegmental feedback during Forward propagation; Ifb-Bwd, intersegmental feedback during backward propagation; MDN, Mooncrawler Descending Neuron; AMB, Ascending Moonwalker-like Backward neuron; *A*(*n*), the *n*-th abdominal neuromere; A(*n* + 1), the (*n* + 1)th abdominal neuromere; SEZ, the subesophageal zone. A, anterior; P, posterior.

The segmental architecture of the VNC suggests that the neural networks involved in larval locomotion should include three types of coordination: intrasegmental, intersegmental, and descending/ascending coordination (Figure 5). These coordination mechanisms ensure the synchronized contraction of muscle within segments and the propagation of activity across segments, facilitating efficient crawling.

In intrasegmental coordination (Figures 5A, B), specific interneurons within a neuromere regulate the activity of motor neurons in the same neuromere (Figure 5B). One type of inhibitory premotor interneuron PMSIs (period-positive median segmental interneurons) plays a crucial role in this process (Kohsaka et al., 2014). PMSIs suppress motor neurons and confine their burst duration, thereby influencing the crawling speed. Additionally, a GABAergic interneuron iIN1 (inhibitory interneuron 1) inhibits motor neurons that target transverse muscles (Zwart et al., 2016). This interneuron contributes to the coordination between earlier and later-recruited motor pools, influencing the timing of muscle contractions. Another GABAergic interneuron called GDL (GABA-positive dorsal lateral neuron) innervates premotor interneuron A27h and is involved in regulating the crawling speed (Fushiki et al., 2016). Finally, EL neurons, (Eve-positive lateral neurons) which are contralateral excitatory interneurons, facilitate the bilateral coordination of muscle contraction (Heckscher et al., 2015). These intrasegmental coordination mechanisms ensure the proper timing and duration of muscle contraction within individual segments.

In addition to intrasegmental coordination, intersegmental coordination is crucial for larval locomotion (Figures 5C, D). This coordination involves interaction between different neuromeres in the VNC to ensure the propagation of peristaltic waves along the body axis. Several interneurons have been identified to be involved in intersegmental coordination, including Ifb-Fwd (Intersegmental feedback during Forward propagation), Ifb-Bwd (Intersegmental feedback during Backward propagation), and Canon neurons (Figure 5D; Fushiki et al., 2016; Kohsaka et al., 2019; Hiramoto et al., 2021). Ifb-Fwd and Ifb-Bwd are direction-specific interneurons, meaning they are selectively activated during either forward or backward crawling. Ifb-Fwd is activated during forward crawling but not in backward crawling; in contrast, Ifb-Bwd is activated during backward crawling but not forward crawling. Ifb-Fwd and Ifb-Bwd share postsynaptic premotor interneurons and are involved in coordinating the contraction of transverse muscles (Kohsaka et al., 2019). Canon neurons are another type of intersegmental interneurons that innervate multiple neighboring neuromeres. They are specifically activated during backward crawling and are involved in the relaxation of muscles (Hiramoto et al., 2021).

Ascending and descending neurons also play a role in larval crawling (Figures 5E, F). MDNs (Mooncrawler Descending Neurons) are located in the brain and send descending axons into the VNC (Carreira-Rosario et al., 2018). Activation of MDNs induces backward crawling, suggesting that they serve as a command neuron for initiating backward locomotion. AMB (Ascending Moonwalker-like Backward) neurons mediate sensory stimuli that trigger backward crawling and interact with MDNs (Omamiuda-Ishikawa et al., 2020). A recent brain-wide mapping study reveals the entire projection from the brain to the VNC (Winding et al., 2023). The report shows that only a small population of premotor circuit neurons are targeted by descending neurons, indicating that descending neurons likely play a role in switching behavior patterns rather than controlling individual motor neurons.

While significant progress has been made in characterizing interneurons involved in larval locomotion, our understanding of the motor circuits is still far from complete. Further detailed characterization of individual interneurons and their specific roles will be essential for unraveling the circuit mechanisms underlying motor control. Moreover, potential evolutionary homologs of some larval interneurons have been found in vertebrates (Kohsaka et al., 2014; Heckscher et al., 2015), which highlights the relevance of studying larval motor circuits in understanding the operational mechanisms of neural circuits across different species.

### Imaging-based approaches to analyze population dynamics of neural activity

The spatiotemporal activity patterns in the CNS instruct the coordinated movements of the whole animal body. Optical imaging of neural activity with genetically encoded probes has paved the way to analyze the dynamic activity patterns within the neural circuit (Miyawaki et al., 1997; Zhao et al., 2011; Ohkura et al., 2012; Chen et al., 2013; Dana et al., 2016; Bando et al., 2019; Inoue et al., 2019; Swanson et al., 2022). To detect calcium signals from neurons and synapses through long-term imaging, fast scanning speed and low photobleaching are desirable. These requirements have been fulfilled by spinning-disk confocal microscopy and light-sheet microscopy (Ahrens et al., 2013; Keller et al., 2014; Chhetri et al., 2015; Lemon et al., 2015; Wan et al., 2019; Yoon et al., 2019). These techniques have revolutionized the study of neural activity patterns in a wide area of the CNS and their relationship to animal behaviors, including larval locomotion.

### Population circuit activity patterns for locomotion

One advantage of using the CNS of fly larvae is that the isolated preparation exhibits spontaneous motor-related activity called fictive motion. The use of isolated CNS enables researchers to reliably monitor calcium signals with high resolution. Isolated CNS has been employed to investigate neural activity in the spontaneous fictive motion in many animals, including vertebrates (Grillner and Jessell, 2009; Garcia-Campmany et al., 2010; Grillner and Kozlov, 2021; Gosgnach, 2023) such as zebrafish (Kyriakatos et al., 2011), tadpoles (Roberts et al., 2010), turtles (Paul and Stein, 2018), rats (Falgairolle and Cazalets, 2007), and mice (Falgairolle and O’Donovan, 2019), but also invertebrates (Marder and Calabrese, 1996; Harris-Warrick, 2010; Selverston, 2010; Büschges et al., 2011; Mantziaris et al., 2020) such as leeches (Kristan et al., 2005), crayfish (Stein et al., 2022), locusts (Mantziaris et al., 2020), stick insects (Bidaye et al., 2018), crabs and lobsters (Marder and Bucher, 2007), and cockroaches (Ayali et al., 2015). By analyzing the spatiotemporal activity patterns during fictive larval behavior, the dynamics of motor circuits can be examined in detail. Studies have revealed the presence of propagating waves of neural activity in the brain that mirror the wave of contractions in the musculature (Lemon et al., 2015). Lesion experiments have shown that different regions in the VNC are capable of initiating fictive forward and backward crawling (Pulver et al., 2015; Jonaitis et al., 2022). Moreover, the thoracic segments have been identified as critical for generating the asymmetric activity of the VNC during bending behavior (Berni, 2015).

To gain insights into the circuit mechanisms responsible for generating the global spatiotemporal activity patterns, it would be informative to analyze synaptic activity across the entire CNS. However, the sheer number of synapses in the CNS poses a challenge for synapse-level analysis. To overcome this problem, a statistical method has been developed to extract bouton-like structures from pan-neuronal calcium imaging data of the fly CNS (Fukumasu et al., 2022). Based on a simulated annealing method developed in statistical physics, thousands of boutons have been extracted from calcium imaging data of the VNC during fictive forward and backward crawling. These data have revealed that the neural networks involved in crawling behavior exhibit a scale-free property, which is commonly observed in neural networks across different organisms, from nematodes to humans (Barabási and Albert, 1999; Eguíluz et al., 2005; Stam and Reijneveld, 2007). According to the graph theory, if the network connection is random, the number of connections from each neuron follows the Poisson distribution (Erdös and Rényi, 1959). This distribution can be approximated by the Gaussian distribution, and there are few neurons that have a large number of connections with other neurons. On the other hand, the scale-free network has distinct statistical properties from random networks. It possesses a few numbers of neurons that connect with a great number of other neurons. It is possible that the minority cell groups act as “hub neurons” that orchestrate the entire network through the rich synaptic connection (Sporns et al., 2007; Sporns, 2018). The cellular identity of these hub neurons and the relevant network architecture in larval motor circuits will be revealed by single-cell level studies using genetics and connectomics. These approaches can provide detailed insights into the global structure of larval motor circuitry.

## Exploration of environments

### Quantification of larval exploration

Fly larvae on a surface exhibit an exploration behavior characterized by a temporal sequence of crawling, stopping, bending, and turning (Green et al., 1983; Figure 2D). Analyzing the kinematic patterns of larval exploration involves assessing the position and posture of a larva. The position of the larva’s centroid over time provides information about the speed and direction of crawling movement (see section “Kinematics of larval locomotion”). From time to time, larvae bend their body at the middle segments laterally, and in some cases, they change their direction to crawl (Lahiri et al., 2011). Furthermore, larvae perform head-sweeping motions, which involve the movement of photosensors (the Bolwig organs) and chemosensors (the dorsal organs) scanning their environments (Gomez-Marin et al., 2011; Gershow et al., 2012; Kane et al., 2013; Gepner et al., 2015). Bending, turning, and sweeping can be analyzed by measuring the posture of the larva.

Advancements in computer vision technology have significantly facilitated the quantitative analysis of larval position and posture. Prior to the prevalent use of computer-aided tracking methods in the research fields, researchers had to manually identify and record feature points, including the centroid, in each frame of the recorded videos, which was a laborious and time-consuming process. To overcome these challenges, custom-made software tools have been devised based on the specific image properties and kinematic information required by researchers (Gomez-Marin et al., 2011, 2012; Lahiri et al., 2011; Gershow et al., 2012; Gepner et al., 2015, 2018), MAGAT (Gershow et al., 2012; Gepner et al., 2015, 2018), MWT (Swierczek et al., 2011; Ohyama et al., 2013), and FIMTrack (Risse et al., 2017). These tracking systems utilize machine vision techniques to automatically identify individual larvae in images and extract various geometric features such as the larva’s center of mass position, body midline, locomotion speed, and positions of the head and tail. In recent years, deep learning technology in artificial intelligence has been employed to further enhance larval tracking and behavior analysis. These tools can track body parts without the need for specific markers, reduce the calculation cost, and improve behavior classification accuracy [DeepLabCut (Mathis et al., 2018), TRex (Walter and Couzin, 2021), and LabGym (Hu et al., 2023)]. These advancements in computer vision and deep learning technology have greatly expedited the analysis of larval locomotor behaviors and exploratory strategies.

### Neural circuits for exploration

The use of computer-aided semi-automated quantification methods has facilitated detailed examinations of the neural mechanisms and statistical properties underlying larval exploration. Extensive research has focused on investigating the neural circuits responsible for phototaxis, thermotaxis, and chemotaxis (Luo et al., 2010; Kane et al., 2013; Gepner et al., 2015; Klein et al., 2015). Furthermore, larval responses to mechanical stimuli such as nociceptive mechanical stimuli and air currents have been explored, leading to the identification of relevant neural circuits (Ohyama et al., 2013, 2015; Jovanic et al., 2016; Masson et al., 2020). Through these studies, fly larvae have served as a valuable model for investigating the neural circuitry mechanisms involved in exploration guided by various sensory modalities.

Interestingly, even under uniform conditions, fly larvae exhibit characteristic exploration behavior. This behavior has been found to resemble a Levy walk (Sims et al., 2019), which is characterized by clusters of short straight paths interspersed with long jumps (Sims et al., 2008). Levy-walk-like behavior has been observed in a wide range of animals, from insects to humans, and is thought to be an optimal strategy for searching for resources in natural environments (Viswanathan et al., 1999; see also James et al., 2011; Reynolds, 2018). Because of the Levy-walk-like characteristics, the kinematics of larval crawling appears to have evolved to optimize food searching. Importantly, the Levy walk behavior in larvae does not require sensory neurons or brain activity, indicating that it is generated by intrinsic, autonomous neural activity in the thoracic and abdominal neuromeres (Sims et al., 2019). As for the origin of the intrinsic control of larval exploration, a theoretical analysis predicts that the body of fly larvae *per se* can generate stochastic crawling patterns in a chaotic manner (Loveless et al., 2019). In conclusion, the quantification of fly larval behavior using computer-aided methods offers valuable insights into the exploration behaviors generated intrinsically or guided by environmental cues. The study of larval exploration provides a unique opportunity to reveal the neural mechanisms underlying complex behaviors and their evolutionary adaptations.

## Interspecific comparison

The study of the larval behaviors and neural circuits in *Drosophila melanogaster* as a model organism (Bellen et al., 2010) has provided a strong foundation for understanding the neural mechanisms of motor control. This deep understanding of a single species can serve as a valuable starting point for exploring interspecific diversity and adaptations in behavior.

Animals have evolved behaviors that are specifically tailored to meet their survival needs within their unique habitats. This includes adaptations in motor patterns and the underlying neural circuits to adapt. It would therefore be reasonable to assume that the larval motor system in fruit flies has also undergone evolutionary adaptations to suit their respective environments. Given the relatively extensive knowledge of the neural circuits and locomotion kinematics in fly larvae, they can provide an insightful model system for studying how animal behaviors adapt to different environments.

The genus *Drosophila*, which includes the widely studied species *Drosophila melanogaster*, contains more than 1,600 species (O’Grady and DeSalle, 2018) with habitats spread across the globe. By comparing the behavior and neural mechanisms of closely related species within this genus, it will become possible to gain insights into the adaptation of behaviors to different environments. Numerous species have been collected and are available through fly stock centers worldwide, such as the Bloomington *Drosophila* Stock Center in the US, the Vienna *Drosophila* Resource Center in Austria, and the Kyoto *Drosophila* Stock Center and Kyorin-Fly in Japan.

**FIGURE 6:**
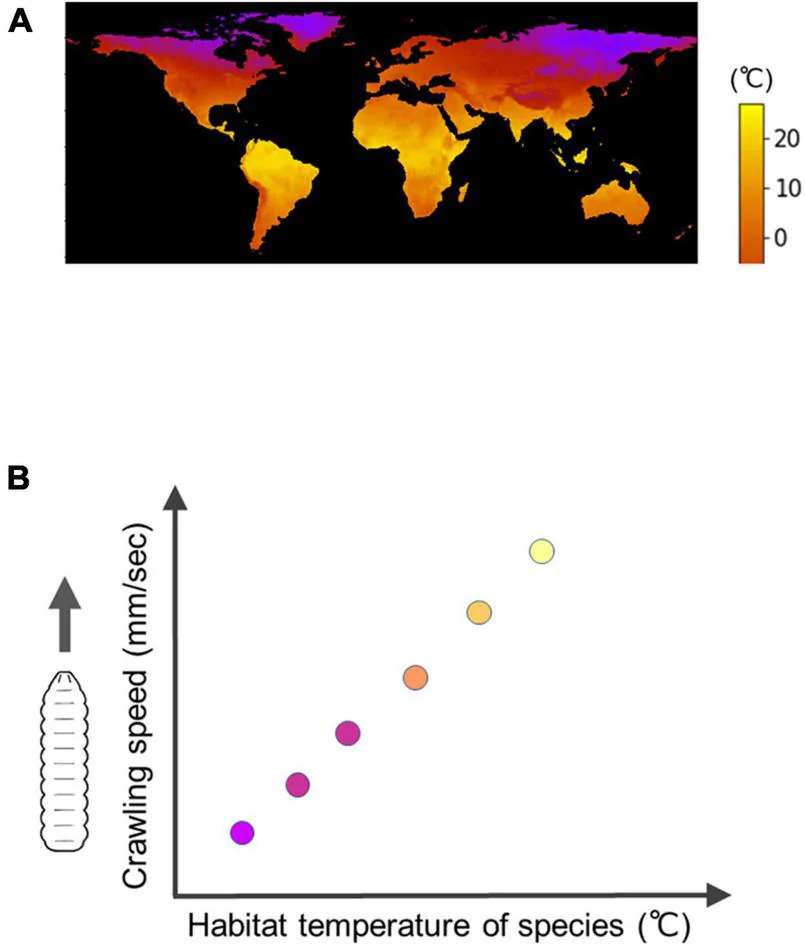
Interspecific diversity in the speed of *Drosophila* larval crawling. **(A)** A map of minimum temperature in the world (Courtesy of Matsuo et al., 2021). **(B)** An interspecific trend is that under the same temperature conditions, fly larvae originally inhabited in cool regions have a slower crawling speed compared to larvae originally inhabited in warm regions, which exhibit a faster crawling speed.

Interspecific comparisons of fly larvae have revealed some of the diversity in biological processes among species within the genus *Drosophila* (Kim et al., 2017; Watanabe et al., 2019; Watada et al., 2020; Matsuo et al., 2021). Regarding larval locomotion, the kinematics of larval crawling exhibit variation, with the speed of crawling showing a positive correlation with the temperature in their respective habitats (Figure 6; Matsuo et al., 2021). By comparing the neural circuits and muscular forces among these closely related species, researchers can reveal how larval locomotion has adapted to different environments.

Consequently, leveraging the wealth of knowledge on *D. melanogaster* and conducting interspecific comparisons within the genus *Drosophila* can provide valuable insights into the diversity of behaviors and adaptations to various environments. These studies contribute to our understanding of principles underlying the adaptation of animal behavior and the evolution of neural circuits.

## Soft robotics

As described above, the analysis of physical properties and neural circuits in living animals has provided great insights into the mechanisms underlying larval locomotion. In contrast to the analytical approaches, a synthetic approach can contribute to understanding the motor system. By synthesizing relevant systems, researchers can gain additional insights into the motor system and explore the effects of perturbing different parameters. This approach allows for hypothesis testing and provides a different perspective on understanding larval locomotion.

Perturbation experiments are commonly employed to investigate the operating principles of a system. Genetic manipulation, for example, enables the perturbation of the physiological status of specific neurons while controlling the environmental conditions that can influence the sensory inputs to animals. Interspecific comparisons can be seen as a study of the effects of environmental perturbations during evolution. In addition to these perturbation strategies, soft robotics has emerged as a powerful tool for manipulating the physical properties of locomoting agents (Webb, 2002; Pfeifer et al., 2012; Trimmer, 2013; Calisti et al., 2017).

Crawling robots inspired by animals have been instrumental in understanding the biological principles underlying animal behavior, including crawling gaits. Distinct crawling gaits are observed in different animals, such as two-anchor (e.g., leeches and caterpillars), peristalsis (e.g., fly larvae), and serpentine (e.g., nematodes) (Alexander, 2006). The mechanisms of these crawling gaits have been explored through experiments with robots along with theoretical approaches and computer modeling (Webb, 2017). For instance, the principle of two-anchor crawling, where the friction coefficient in the forward direction should be smaller than that in the backward direction (Alexander, 2006; Calisti et al., 2017), has been substantiated through soft robots inspired by the moth *Manduca sexta* (Trimmer et al., 2012; Umedachi et al., 2016).

One of the essential characteristics in peristaltic crawling is locomotion speed. To study the speed control mechanisms, researchers have fabricated soft robots inspired by animals exhibiting peristalsis, such as earthworms (Mangan et al., 2002; Boxerbaum et al., 2012) and fly larvae (Wei et al., 2016; Sun et al., 2023). In the case of fly larvae, two examples demonstrate the use of soft robots. First, experimental measurements have shown that fly larvae crawl forward faster than backward direction (Heckscher et al., 2012). By employing a soft robot inspired by fly larvae, researchers revealed that the difference in frictional force between forward and backward movement could influence peristaltic speed (Sun et al., 2023). This observation suggests that an asymmetry in the frictional force, possibly related to the orientation asymmetry of denticle belts (spike-like structures at the bottom of the larval body), might contribute to the direction-specific speed difference. Second, a neuromechanical model predicts that crawling speed should increase with enhanced muscular contraction force (Sun et al., 2022). While inducing spatiotemporal activity mimicking peristaltic waves of motor neurons within the intact fly larval CNS is challenging, soft robots were utilized to test the effect of contraction force on crawling speed. The vacuum-actuated soft robot exhibited a positive correlation between vacuum pressure amplitude and peristaltic crawling speed (Sun et al., 2023). Thus, the use of soft robots opens up new avenues for studying the kinematics of larval locomotion.

It is important to note that soft robots cannot be a perfect replica of soft-bodied animals. Physical properties, such as body shape, material flexibility, contraction force, and surface texture might be simplified in soft robots. Moreover, sensors and feedback mechanisms in soft robots may differ from those in soft-bodied animals. However, soft robots have successfully reproduced animal behavior qualitatively (Pfeifer et al., 2012; Trimmer, 2013; Calisti et al., 2017). One significant advantage of using soft robots is their controllability. For instance, researchers can manipulate the intensity and temporal patterns of contraction forces to study the mechanics of animal movement, which is practically challenging to achieve in experiments with living animals (Sun et al., 2023). Thus, soft robots offer a new strategy for controlling physical properties in moving agents, shedding light on qualitative characteristics of animal locomotion.

In summary, adopting a synthetic approach, including the use of soft robots, provides valuable opportunities to perturb and study various parameters in larval locomotion, enabling a deeper understanding of the underlying mechanisms and kinematics.

## Conclusion

The interplay between neural circuits and the body generates animal behavior. The study of behavior is, to a large part, investigating how neural activity is orchestrated and transformed into muscular contraction forces, muscle movement, and in turn, coordinated motion of the body. *Drosophila* larval locomotion serves as an ideal model for scrutinizing the neuromechanical system underlying animal behavior. The kinematics of larval crawling can be described using a set of parameters, and physical properties such as forces, elasticity, and viscosity have been experimentally measured to understand the dynamics within the framework of classical mechanics. Integrative approaches that combine neural circuits and physical models have enabled the establishment of quantitative neuromechanical models for describing larval locomotion.

Cutting-edge techniques such as connectomics and genetics, along with state-of-the-art imaging methods, have been employed to examine the neural circuits involved in larval locomotion. By studying the population activity within these circuits, researchers have gained insights into how locomotion is regulated during larval exploration. The detailed knowledge about larval locomotion in *D. melanogaster* serves as a foundation for examining interspecific diversity in animal behaviors. In addition to the analytical approach, a synthetic approach utilizing soft robots can provide a further understanding of the operational mechanisms in larval locomotion.

Although several key interneurons have been identified, our knowledge about the neural circuits for larval locomotion is far from complete. Further investigation on the neural networks is necessary to comprehend the circuit mechanisms to realize well-coordinated as well as adaptive larval locomotion. Perspectives for future studies also include exploring interspecific diversity in neural circuits and developing practical soft robots inspired by larvae. While simple linear larval crawling has been successfully reproduced through computer simulations, the mechanisms underlying diverse and adaptive larval locomotion remain unclear. Furthermore, the effects of several complexities in the neuromechanical system, such as non-linearity in the kinematic control, energy dissipation, and coupling between body parts, require further examination.

By adopting interdisciplinary and cooperative approaches, the operating principles of crawling in the tiny 4-mm fruit fly larva will be unraveled. This knowledge can help bridge the gap between neural circuits and physical animal movements and provide a practical framework for studying behavior in a wide variety of animal species.

## Author contributions

HK wrote the manuscript and prepared the figures.
