# Correction: Linking neural circuits to the mechanics of animal behavior in *Drosophila* larval locomotion

**DOI:** 10.3389/fncir.2026.2010536

**Published:** 2026-10-26

**Authors:** Hiroshi Kohsaka

**Affiliations:** 1 Graduate School of Informatics and Engineering, The University of Electro-Communications, Chofu, Tokyo, Japan; 2 Department of Complexity Science and Engineering, Graduate School of Frontier Science, The University of Tokyo, Chiba, Japan

**Keywords:** *Drosophila*, locomotion, motor circuits, kinematics, kinetics, exploration, interspecific diversity

In the published article, numerical estimates for the numbers of muscles, bones, and joints in the adult human body were provided without supporting references. As these numbers can vary depending on anatomical definitions and classifications, the specific numerical estimates have been replaced with a more general description. The original text stated:

“From the neural circuit level [cf. ~86 billion neurons in a human brain (Azevedo et al., 2009)] to the body level (cf. ~640 muscles, ~200 bones, and ~360 joints in an adult human), the degrees of freedom are reduced.”

A correction has been made to the **Introduction**, paragraph 2:

“From the neural circuit level [cf. ~86 billion neurons in the human brain (Azevedo et al., 2009)] to the body level (cf. hundreds of muscles, bones, and joints in the adult human body), the degrees of freedom are reduced.”

The original version of this article has been updated.

